# Supplementary material for: The health and economic burden of respiratory syncytial virus associated hospitalizations in adults
Source: PLoS One. 2020 Jun 11;15(6):e0234235. doi: 10.1371/journal.pone.0234235 (PMC7289360; doi:10.1371/journal.pone.0234235)
Supplement: S1 Table — (DOCX) [file pone.0234235.s001.docx]

**Table S1: Performance of hospital assays (clinician ordered tests) in detecting different respiratory viruses 2012-2016**

Two major public hospitals, the Auckland and Counties Manukau District Health Board hospitals (ADHB and CMDHB), served the populations living in the central, eastern, and southern Auckland region in 2012-2016 and were the study sites of the SHIVERS surveillance. The performance of hospital assays from clinician ordered tests were compared to CDC’s real-time RT-PCR as a gold standard. Only results from assays with a sensitivity greater than 80% and a specificity greater than 95% were included in the SHIVERS study dataset.

| Virus type | Auckland hospital assay | |  | Counties Manukau hospital assay | |
| --- | --- | --- | --- | --- | --- |
|  | Sensitivity (95% CI) | Specificity (95% CI) |  | Sensitivity (95% CI) | Specificity (95% CI) |
| Flu | 96.49 (94.74-97.67) | 99.75 (99.52-99.87) |  | 97.82 (96.43-98.67) | 99.50 (99.29-99.65) |
| RSV | 95.74 (93.84-97.08) | 99.61 (99.32-99.78) |  | 91.38 (88.95-93.32) | 98.18 (97.47-98.69) |
| RV | 94.79 (92.48-96.42) | 99.86 (99.58-99.95) |  | ------------------------ | 85.71 (60.06-95.99) |
| ADV | 87.87 (83.45-91.23) | 99.65 (99.39-99.80) |  | 69.87 (64.56-74.70) | 97.89 (97.14-98.44) |
