# Supplementary material for: The health and economic burden of respiratory syncytial virus associated hospitalizations in adults
Source: PLoS One. 2020 Jun 11;15(6):e0234235. doi: 10.1371/journal.pone.0234235 (PMC7289360; doi:10.1371/journal.pone.0234235)
Supplement: S2 Table — (DOCX) [file pone.0234235.s002.docx]

Table S2: Comparison of tested and non-tested ARI hospitalizations stratified by SARI case definition among adults aged 18 or older in Auckland, New Zealand 2012-2015.

|  | All ARI hospitalizations | | SARI | | | | | non-SARI acute respiratory | | | | |
| --- | --- | --- | --- | --- | --- | --- | --- | --- | --- | --- | --- | --- |
|  | Total | | Tested | | non-tested | | p-value^*^ | Tested | | non-tested | | p-value^*^ |
|  | N | (Col %) | N | (Col %) | N | (Col %) |  | N | (Col %) | N | (Col %) |  |
| Total | 8776 | (100.0) | 3046 | (100.0) | 758 | (100.0) |  | 1554 | (100.0) | 3418 | (100.0) |  |
| Age Group |  |  |  |  |  |  |  |  |  |  |  |  |
| 18-49 | 2117 | (24.1) | 982 | (32.2) | 185 | (24.4) | <0.001 | 385 | (24.8) | 565 | (16.5) | <0.001 |
| 50-64 | 1974 | (22.5) | 754 | (24.8) | 159 | (21.0) | 0.009 | 375 | (24.1) | 686 | (20.1) | 0.059 |
| 65-79 | 2677 | (30.5) | 824 | (27.1) | 212 | (28.0) | 0.917 | 484 | (31.1) | 1157 | (33.9) | 0.519 |
| ≥80 yrs | 2008 | (22.9) | 486 | (16.0) | 202 | (26.6) | <0.001 | 310 | (19.9) | 1010 | (29.5) | <0.001 |
| Sex |  |  |  |  |  |  |  |  |  |  |  |  |
| Female | 4682 | (53.4) | 1673 | (54.9) | 392 | (51.7) | 0.125 | 855 | (55.0) | 1762 | (51.6) | 0.376 |
| Male | 4094 | (46.6) | 1373 | (45.1) | 366 | (48.3) | 0.124 | 699 | (45.0) | 1656 | (48.4) | 0.403 |
| SES^†^ |  |  |  |  |  |  |  |  |  |  |  |  |
| 1 (least deprived) | 931 | (10.6) | 318 | (10.4) | 77 | (10.2) | 0.783 | 171 | (11.0) | 388 | (11.4) | 0.767 |
| 2 | 1216 | (13.9) | 438 | (14.4) | 110 | (14.5) | 0.870 | 227 | (14.6) | 511 | (15.0) | 0.236 |
| 3 | 1335 | (15.2) | 432 | (14.2) | 113 | (14.9) | 0.703 | 225 | (14.5) | 516 | (15.1) | 0.414 |
| 4 | 1258 | (14.3) | 399 | (13.1) | 103 | (13.6) | 0.764 | 220 | (14.2) | 522 | (15.3) | 0.589 |
| 5 (most deprived) | 4036 | (46.0) | 1459 | (47.9) | 355 | (46.8) | 0.903 | 711 | (45.8) | 1481 | (43.3) | 0.100 |
| Ethnicity |  |  |  |  |  |  |  |  |  |  |  |  |
| Maori | 1585 | (18.1) | 560 | (18.4) | 130 | (17.2) | 0.406 | 266 | (17.1) | 629 | (18.4) | 0.063 |
| Pacific | 2552 | (29.1) | 1026 | (33.7) | 246 | (32.5) | 0.858 | 426 | (27.4) | 854 | (25.0) | 0.045 |
| Asian | 811 | (9.2) | 321 | (10.5) | 93 | (12.3) | 0.029 | 119 | (7.7) | 278 | (8.1) | 0.070 |
| European/Other | 3828 | (43.6) | 1139 | (37.4) | 289 | (38.1) | 0.395 | 743 | (47.8) | 1657 | (48.5) | 0.571 |

* Characteristics of tested and non-tested cases were compared using Chi-square tests.

† SES quantified using a small area level measure of household deprivation derived from the national census (NZDep2013). This measure was used to divide the study sample into quintiles with SES 1 as least deprived and SES 5 as most deprived.^28^
