# Supplementary material for: The health and economic burden of respiratory syncytial virus associated hospitalizations in adults
Source: PLoS One. 2020 Jun 11;15(6):e0234235. doi: 10.1371/journal.pone.0234235 (PMC7289360; doi:10.1371/journal.pone.0234235)
Supplement: S3 Table — (DOCX) [file pone.0234235.s003.docx]

Table S3: Comparison of SHIVERS systematic and clinical ordered testing among ARI hospitalizations in adults aged 18 or older in Auckland, New Zealand 2012-2015.

|  | **SHIVERS** | **Col (%)** | **Clinician** | **Col (%)** | **p-value** |
| --- | --- | --- | --- | --- | --- |
| Total | 3220 | (100.0) | 1371 | (100.0) |  |
| Age grp (years) |  |  |  |  |  |
| 18-49 | 1058 | (32.9) | 307 | (22.4) | <0.001 |
| 50-64 | 783 | (24.3) | 341 | (24.9) | 0.267 |
| 65-79 | 892 | (27.7) | 415 | (30.3) | 0.928 |
| ≥80 | 487 | (15.1) | 308 | (22.5) | <0.001 |
| Sex |  |  |  |  |  |
| F | 1779 | (55.2) | 742 | (54.1) | 0.506 |
| M | 1441 | (44.8) | 629 | (45.9) | 0.506 |
| SES* |  |  |  |  |  |
| 1 | 362 | (11.2) | 126 | (9.2) | 0.027 |
| 2 | 482 | (15.0) | 180 | (13.1) | 0.313 |
| 3 | 476 | (14.8) | 181 | (13.2) | 0.153 |
| 4 | 438 | (13.6) | 178 | (13.0) | 0.191 |
| 5 | 1462 | (45.4) | 706 | (51.5) | 0.001 |
| Ethnicity |  |  |  |  |  |
| Māori | 609 | (18.9) | 215 | (15.7) | 0.009 |
| Pacific | 994 | (30.9) | 457 | (33.3) | 0.100 |
| Asian | 290 | (9.0) | 150 | (10.9) | 0.042 |
| European/Other | 1327 | (41.2) | 549 | (40.0) | 0.462 |
| Case definition |  |  |  |  |  |
| SARI | 2641 | (82.0) | 399 | (29.1) | <0.001 |
| non-SARI | 579 | (18.0) | 972 | (70.9) | <0.001 |
| ICU |  |  |  |  |  |
| Not admitted | 3124 | (97.0) | 1328 | (96.9) | 0.779 |
| Admitted | 96 | (3.0) | 43 | (3.1) | 0.779 |
| LOS |  |  |  |  |  |
| Mean (IQR) | 4.1 | (2-5) | 4.6 | (2-6) | 0.001 |
| Primary ICD-10 codes (most common) |  |  |  |  |  |
| Influenza (J10) | 356 | (11.1) | 162 | (11.8) | 0.224 |
| Viral pneumonia (J12) | 71 | (2.2) | 39 | (2.8) | 0.338 |
| Pneumonia due to Step pneumonia (J13) | 131 | (4.1) | 50 | (3.6) | 0.414 |
| Pneumonia unspecified (J18) | 603 | (18.7) | 220 | (16.0) | 0.078 |
| Unspecified ALRI (J22) | 327 | (10.2) | 154 | (11.2) | 0.363 |
| COPD (J44) | 508 | (15.8) | 245 | (17.9) | 0.679 |
| Asthma (J45) | 240 | (7.5) | 109 | (8.0) | 0.691 |

*SES quantified using a small area level measure of household deprivation derived from the national census (NZDep2013). This measure was used to divide the study sample into quintiles with SES 1 as least deprived and SES 5 as most deprived.28
